# Eco-Friendly In-Situ Zwitterionization of Stent Metals with Phosphonic Sulfobetaine Copolymers for Durable Antibiofouling and Hemocompatibility

**DOI:** 10.1021/acs.langmuir.6c02010

**Published:** 2026-08-10

**Authors:** Irish Valerie Maggay, Gian Vincent Dizon, Deng-Shin Chen, Jen-Yi Lee, Antoine Venault, Chung-Jung Chou, Jie Zheng, Yung Chang

**Affiliations:** † R&D Center for Membrane Technology and Department of Chemical Engineering, 34900 Chung Yuan Christian University, Chung-Li, Taoyuan, 32023, Taiwan; ‡ Department of Biomedical Engineering and Chemical Engineering, 12346 University of Texas at San Antonio, San Antonio, Texas 78249, United States

## Abstract

Although stainless steel (SS) is widely used for cardiovascular stents, its surface is prone to undesirable biological interactions that contribute to thrombosis, infection and restenosis. In this study, zwitterionic copolymers composed of poly­(vinylphosphonic acid) (PVPA) and poly­(4-vinylpyridine propylsulfobetaine) (P4VPPS) were successfully synthesized, structurally characterized, and grafted onto SS surfaces via a green, water-based approach. Dynamic vapor sorption and zeta potential measurements revealed hydration characteristics governed by the balance between phosphonic acid and zwitterionic segments. Among the copolymers, VPA/4VPPS ratio of 70/30 (VPS70) exhibited the most favorable hydration behavior, and near neutral surface charge, which collectively resulted in superior antifouling and bioinert performance. The VPS70-grafted SS surface effectively suppressed adhesion of fibroblast cells (reduced by 83.7%), *Escherichia coli* (reduced by 68.8%), and blood components (reduced by 78.8%), while maintaining good cytocompatibility (88.3% cell viability), nonhemolytic behavior (0% hemolysis), and without affecting blood coagulation (PT and APTT values comparable to the control). Notably, VPS70 retained its bioinert properties after steam sterilization at 121 °C, demonstrating superior thermal stability over sulfobetaine methacrylate-based systems. These results establish VPS70 as a promising grafting material for cardiovascular SS-based stents capable of minimizing blood cell and bacterial adhesion, reducing dependence on antithrombotic therapy, and mitigating restenosis. Moreover, this water-based grafting method offers a simple and practical way to modify SS surfaces used in blood-contacting medical devices.

## Graphical abstract

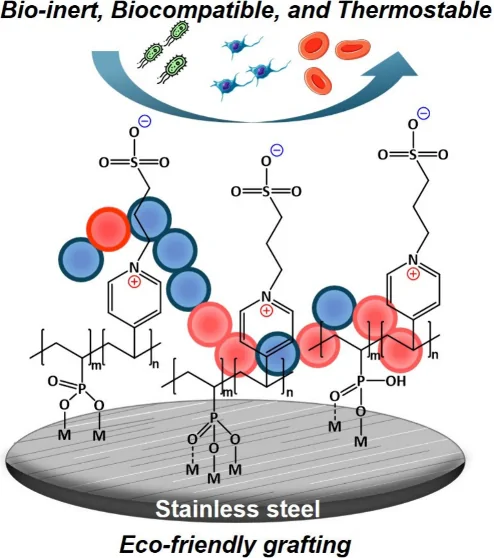

## Introduction

Cardiovascular diseases (CVD) are among the leading causes of death worldwide, accounting for nearly one-third of all global deaths, and their incidence continues to rise.^1–4^ It is projected that by 2030, the annual death toll from CVD worldwide will reach as high as 23 million.^5^ Among these conditions, coronary artery disease (CAD) is one of the most prevalent and is characterized by the accumulation of atherosclerotic plaque within the coronary arteries.^4^ Plaque buildup can lead to stenotic plaques that significantly narrow arteries (>50% stenosis) and restrict blood flow, or nonstenotic plaques that cause minimal obstruction (<50% stenosis) but still carry a high risk of rupture and acute cardiovascular events.^4,6,7^ Management of stenotic lesions often require revascularization via percutaneous coronary intervention (PCI) or coronary artery bypass grafting (CABG). While CABG is generally reserved for complex multivessel diseases, PCI is often favored for patients with acute coronary syndrome (ACS).^8,9^ PCI involves advancing a balloon-tipped catheter to the stenotic artery, a procedure initially known as percutaneous transluminal coronary angioplasty (PTCA) or simply angioplasty.^4^ In modern practice, PCI is commonly performed with stent placement, which acts as a permanent scaffold that maintains vessel patency, restores blood flow, and reduces the probability of restenosis.^10–12^

The earliest stents were bare-metal stents (BMS) made of stainless steel (SS).^13^ However, BMS are prone to in-stent restenosis (ISR) due to endothelial cell growth, causing loss of functionality.^14^ To overcome this, drug-eluting stents (DES) that coat the stent with drugs to inhibit cell proliferation have been developed.^15–18^ In order to avoid the permanent presence of metal stents in the body, bioresorbable vascular scaffolds (BRS) that gradually degrade over time have been developed in recent years.^19^ While promising, clinical studies revealed higher rates of cardiac death, myocardial infarction, and target lesion revascularization compared to DES.^19,20^ The current challenges faced by vascular stents include the need for long-term administration of antithrombotic medications to prevent clotting reactions upon contact with the stent and the phenomenon of ISR caused by endothelial cell growth on the stent after prolonged use.^10,14,21^ However, the long-term efficacy of DES is limited by the finite duration of drug release.^22^ Given the persistent risks of thrombosis and restenosis with current stent technologies, there remains a need for more effective and durable modification strategies.

A promising approach to overcome these challenges is the surface modification of stents with bioinert materials,^23,24^ with zwitterionic polymers being particularly attractive for their capacity to prevent the adhesion of proteins, cells, and platelets.^25–29^ By disrupting the initial interfacial interactions, zwitterion can effectively suppress the cascade of biological responses associated with thrombosis and restenosis.^25,27,30–32^ Recent years have seen the development of various zwitterionic materials such as phosphobetaine (PB)^33^ sulfobetaine (SB),^34–36^ and carboxybetaine (CB).^37–39^ Among these, sulfobetaine methacrylate (SBMA) has been widely investigated due to its relatively low-cost, ease of synthesis, stability, which have supported its broad application.^40–43^ In particular, poly­(sulfobetaine methacrylate) (PSBMA) has been shown to provide excellent hemocompatibility and effectively inhibit blood clotting reactions.^44^ However, SBMA-based polymers are susceptible to thermal degradation at elevated temperature because of ester bond cleavage.^45,46^ We have recently introduced a copolymer based on poly­(4-vinylpyridine propylsulfobetaine) (4VPPS), designed to exhibit both heat tolerance and antifouling performance, synthesized via the quaternization of 4-vinylpyridine with propane sultone.^47,48^ This copolymer has been shown to resist HT1080 cell adhesion even after steam sterilization, with no attachment observed even after 5 days of incubation.^48^ These findings suggest that the copolymer has the potential to mitigate revascularization, and it can retain its antifouling properties even at elevated temperatures, an essential requirement for biomedical applications.

While the development of thermally stable zwitterionic polymers is essential in biomedical applications, the method by which they are immobilized on metallic substrates is equally critical. Coating offers a simple approach,^49^ but it relies only on physical interactions that are unstable under physiological conditions.^32^ Instead, stable grafting methods are required, in which the bioinert polymer is chemically anchored to the metallic substrate through covalent.^50^ For SS or titanium-based stents, anchoring agents such as silane-based,^51–53^ dopamine-based,^52,54,55^ and phosphonate or phosphonic acid–based^56–60^ have been employed to establish covalent bonds that enable the effective grafting of bioinert materials onto the metal surface. A key drawback of silane layers lies on its susceptibility to the hydrolytic breakdown of Si–O–Si bonds.^61^ Previous investigations have challenged the classification of polydopamine (PDA) as a true polymer, instead characterizing it as a disordered aggregate of dopamine monomers and oligomers held together by weak noncovalent interactions, including hydrogen bonding, charge transfer, and π–π stacking.^62,63^ This poorly defined structure makes PDA prone to uncontrolled aggregation which can impact the stability of the surface modification. Meanwhile, phosphonic acids contain a phosphoryl group (P = O) and two hydroxyl groups (P–OH), providing three oxygen atoms that can coordinate with surface metal centers. Through these sites, phosphonic acid derivatives are able to form multidentate M_(metal)_–O-P linkages with the native oxide layer of metal substrates, enabling monodentate, bidentate, or tridentate binding depending on the interaction mode.^56,58,59,64^ Additionally, these phosphate-based anchoring agents function as corrosion inhibitors,^65,66^ protecting stents from degradation in the harsh environment of blood, electrolytes, and oxidative stress,^67^ enhancing their long-term stability. Moreover, the review paper by Boissezon et al. reported that phosphonic acids can bind strongly to several metal oxide surfaces such as titania, alumina, zirconia, silica, and zinc oxide, implying that this anchoring agent provides versatility.^68^

Collectively, these attributes make vinyl phosphonic acid (VPA) a compelling anchoring component for next-generation zwitterionic modifications on metal substrates. By incorporating VPA into the polymer backbone, strong multidentate M–O-P coordination with various metal substrates can be achieved by ensuring strong anchoring while maintaining a hydrated, nonfouling surface. Demonstrating the clinical relevance of this approach requires evaluation in a system capable of maintaining performance under sterilization and blood-contact conditions. For this purpose, poly­(vinylphosphonic acid)-*co*-poly­(4-vinylpyridine propylsulfobetaine) P­(VPA-*co*-4VPPS) was selected as a model grafting system as it combines the strong anchoring capability of VPA with the thermal stability and bioinert properties of 4VPPS. This design aims to overcome the limitations associated with current stent coatings, including thrombosis, in-stent restenosis, and hydrolysis-induced loss of functionality. In keeping with sustainable goals, the synthesis and grafting processes were achieved without toxic solvents, offering an environmentally responsible route to antifouling surface modification. Comprehensive characterization of P­(VPA-*co*-4VPPS) copolymers (with varying molar ratios) to shed light on its chemistry, bulk properties, and surface/interfacial properties. Then, grafting onto SS was conducted under aqueous environment. Subsequently, grafting onto SS was carried out under aqueous conditions. Bioinert properties of were evaluated using human fibrosarcoma cells, fluorescent *Escherichia coli (E. coli)*, and human whole blood through cytotoxicity, hemolysis, and clotting time assays to assess biocompatibility. Together, these results demonstrate a green surface-modification strategy that offers a promising pathway for the design of next-generation stents.

## Experimental Methods

### Materials

4-Vinylpyridine (4VP), sodium chloride (NaCl), and deuterium oxide (D_2_O) for NMR analysis were purchased from Sigma-Aldrich. 1,3-Propane sultone (1,3-PS) was obtained from Taiwan Hopax Chemicals, while vinylphosphonic acid (VPA) was supplied by Tokyo Chemical Industry. Ammonium persulfate (APS) was purchased from Showa Chemical Industry. Deionized water (DI water) was prepared using an SG Ultraclear Basic purification system. SS substrates were provided by Cheng Yun Precision Machinery (Taiwan). Whole blood used for biocompatibility testing was obtained from MacKay Memorial Hospital (Taipei). Genetically modified *E. coli* expressing green fluorescent protein was acquired from the Bioresource Collection and Research Center (Hsinchu, Taiwan).

#### Synthesis of 4VPPS Monomer

Scheme 1a illustrates the spontaneous ring-opening reaction of 4VPPS. To prepare the monomer, 4VP and 1,3-PS were separately dissolved in acetone at a solid content of 20 wt %, with the molar ratio fixed at 1:1.2. The 1,3-PS solution was placed in a round-bottom flask, and the 4VP solution was added dropwise under magnetic stirring. The solution was stirred at room temperature for 24 h. After completion, the product was collected by vacuum filtration and washed twice with acetone to remove unreacted species. The resulting 4VPPS monomer was freeze-dried to remove residual solvent and stored at 4 °C.

**Scheme 1:**
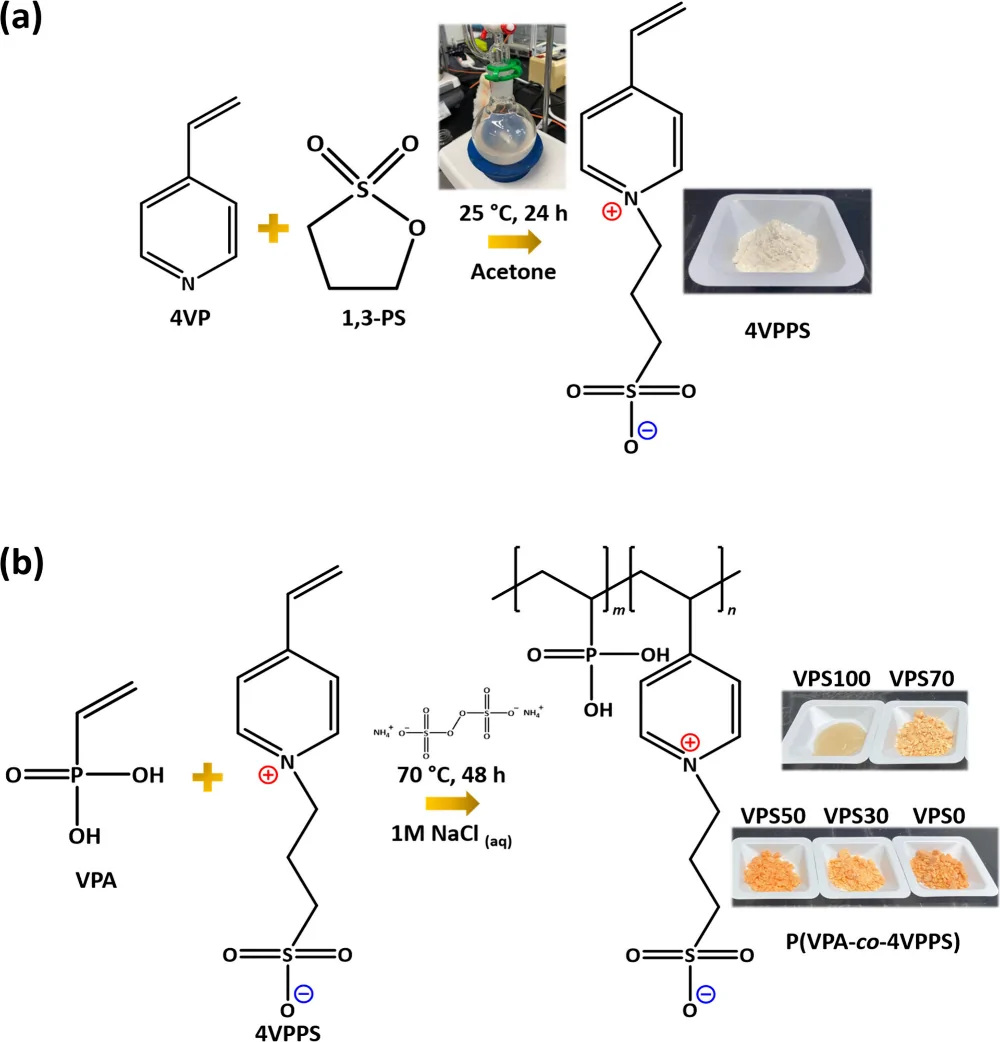
Synthesis of (a) 4VPPS Monomer and (b) P­(VPA-*co*-4VPPS)

#### Polymerization of P­(VPA-*co*-4VPPS) copolymers

The copolymers were synthesized by free radical polymerization (Scheme 1b) with varying feed ratios of VPA and 4VPPS. VPA, 4VPPS, and the initiator APS were dissolved in 1 M NaCl aqueous solution at the ratios listed in Table 1. The resulting formulations were designated as VPSx, where *x* denotes the theoretical molar ratio of VPA. The solution was purged with N_2_ for 30 min and then polymerized in a 70 °C oil bath with stirring for 48 h. After completion, the solution was cooled in an ice bath to terminate the reaction. The copolymer solution was then slowly added dropwise into DI water to precipitate the copolymer and remove unreacted monomers. The final product was obtained by freeze-drying and stored for further use. VPS0, VPS30, VPS50, and VPS70, VPS100 were obtained as powders, whereas VPS100 was obtained in liquid form.

**Table 1:** Comparison of Theoretical and Actual Molar Compositions of the Copolymer with Corresponding GPC Analysis

	**Theoretical molar ratio** (mol %)		**Actual molar composition** **(mol %)**		**GPC analysis**	
**Copolymer ID**	**VPA**	**4VPPS**	**PVPA**	**P4VPPS**	**Molecular weight (kDa)**	**Polydispersity index (PDI)**
**VPS100**	100	0	100	0	0.7	1.5
**VPS70**	70	30	37.8	62.2	29.3	2.4
**VPS50**	50	50	29.6	70.4	61.0	3.6
**VPS30**	30	70	20.1	79.9	88.6	3.3
**VPS0**	0	100	0	100	113.0	3.3

#### Characterization of the 4VPPS and P­(VPA-co-4VPPS) Copolymers

The chemical structures of the monomer and copolymers were examined using ^1^H Nuclear Magnetic Resonance (NMR) spectroscopy. For monomer analysis, 20 mg of 4VPPS was dissolved in 1.3 mL of D_2_O. As for the copolymers: 100 μL of VPS100 was dissolved in 1.3 mL of D_2_O, while 20 mg of VPS0, VPS30, VPS50, and VPS70 copolymers were each dissolved in 1.3 mL of 1 M NaCl solution in D_2_O. Spectra were acquired using a Bruker Avance III HD-600 spectrometer, and the data was processed using MestreNova software. Fourier Transform Infrared (FT-IR) spectroscopy analysis was performed to identify the characteristic functional groups of the samples. Prior to testing, KBr and the samples were mixed at a 100:1 ratio using an agate mortar and pestle, followed by freeze-drying to remove residual moisture. The resulting powders were pressed into 3 mm discs and analyzed on a Jasco FT-IR-4700 spectrometer to obtain the polymer absorption spectra, and spectra were collected in transmission mode (4 cm^–1^ resolution, 32 scans). The molecular weight (M_w_) and polymer dispersity index (PDI) of the samples were determined by gel permeation chromatography (GPC). Samples were prepared by dissolving the samples in 0.2 M NaNO_3_ aqueous solution at a concentration of 3 mg/mL. GPC analysis was conducted on a Shimadzu RID-20A system equipped with a Shodex SB-803 HQ column, using a flow rate of 0.10 mL/min. The M_w_ were calculated based on calibration with polystyrene standards. The water absorption capacity of the copolymer was evaluated using a dynamic vapor sorption analyzer (TA Q5000 SA). Samples (3–5 mg) were first freeze-dried to remove residual moisture before loading into the instrument. Measurements were conducted at 37 °C, starting with 0% relative humidity (RH) for 30 min to stabilize the system. RH was then increased to 98% and held for 2 h to allow water absorption, followed by decrease to 0% RH for 2 h to induce desorption. This hydration ratio was calculated according to the following eq 1:

1 $$Hydration\;ratio\;\left(\%\right)=\frac{W_{98\%}-W_{polymer}}{W_{polymer}}\times 100\%$$

where *W*_98%_ represents the weight of the water absorbed at 98% RH, and *W*_*polymer*_ denotes the initial dry weight of the polymer. For zeta potential measurements, the polymer was dissolved in 1 M NaCl aqueous solution to obtain a 10 mg/mL solution. Particle size and zeta potential were then determined using a dynamic light scattering and zeta potential analyzer (Malvern Zetasizer Nano ZS90).

#### Copolymer Grafting onto SS

Prior to surface modification, SS discs (Ø1 cm) were cleaned by ultrasonication in ethanol and DI water, 30 min each, repeated 3x. The cleaned discs were then dried in an oven at 60 °C for 24 h. Then, the disks were placed into a UV ozone cleaning chamber for 20 min to remove surface impurities. The copolymer was dissolved in 1 M NaCl at a concentration of 40 mg/mL. The SS discs were transferred into a 24 well plate and 1 mL of the copolymer solution were added to each well. Subsequently, the samples were incubated at 60 °C for 48 h to facilitate grafting. Once the reaction was completed, the modified SS discs were washed with 1 M NaCl to remove loosely adhered copolymer followed by DI water. The samples were then dried at room temperature for at least 1 day.

#### Surface Wettability Measurements of the SS

Surface hydrophilicity of SS was assessed by dynamic water contact angle (WCA) measurements using a contact angle analyzer (DataPhysics OCA 15EC). A 4 μL droplet of DI was carefully dropped onto the sample surface and the contact angle was recorded for 3 min.

#### Bioinert Evaluation of the SS

##### HT1080 Cell Adhesion Test

Cell adhesion measurements were evaluated using HT1080 cells. Before cell seeding, the modified SS discs were transferred to a sterile tissue culture polystyrene (TCPS) multiwell plate and incubated with phosphate-buffered saline (PBS) overnight at 37 °C. The PBS was then removed, and the discs were rinsed once with fresh PBS before adding 1 mL of HT1080 cell suspension (50,000 cells/mL) to each well. The samples were incubated for 24 h at 37 °C under 5% CO_2_. After incubation, the samples were gently washed 3x times with PBS to remove nonadherent cells, and 1 mL of 2.5 wt.% glutaraldehyde solution was added on the samples to fix the cells on the SS discs. Following fixation, the samples were rinsed again with PBS, and cell adhesion was observed using a fluorescence microscope (Nikon ECLIPSE 80I).

##### Whole Blood Cell Adhesion Test

Similarly, prior to the whole blood adhesion test, grafted SS samples were incubated in PBS at 37 °C overnight. After removing the PBS, the discs were rinsed once with fresh PBS before adding 1 mL of human whole blood to each sample. The samples were then incubated at 37 °C with shaking at 100 rpm for 1 h. The blood was then removed, and the samples were washed 3x with PBS before being transferred to a fresh TCPS plate and rinsed 3 additional times, and 1 mL of 2.5 wt.% glutaraldehyde solution was added and incubated at 37 °C for 1 h. The samples were subsequently rinsed with PBS, and blood cell adhesion was observed using a confocal laser scanning microscope (CLSM, Nikon A1R).

##### Bacteria Adhesion Test

Bacterial adhesion was evaluated using green fluorescent protein-expressing *E. coli* (GFP *E. coli*). The discs were placed in sterile TCPS multiwell plates and presoaked in PBS overnight at 37 °C. After removing PBS, the samples were rinsed once with PBS, and 1 mL of freshly prepared bacterial suspension (1 × 10^9^ cells/mL) was added. The samples were transferred into an oven set at 37 °C and 100 rpm for 24 h, with the bacterial suspension replaced every 12 h. Following incubation, the discs were washed 3x with PBS, followed by fixation using glutaraldehyde. Bacterial adhesion on the discs surfaces was examined using a fluorescence microscope. The detailed protocol of the bacteria culture is described in a previous work.^69^

##### Thermostability Test

The thermal stability of the grafted coatings was evaluated by subjecting the samples to steam sterilization. VPS70-grafted SS, virgin SS, and PSBMA gel (control) were autoclaved in a Speedy autoclave (Tomin, Japan) at 121 °C for 1 h under high-pressure steam. Immediately after sterilization, human whole blood adhesion tests were conducted following the procedure described in the previous section to assess the stability and bioinert properties of the coatings after thermal treatment.

##### Biocompatibility Tests

The biocompatibility of the modified SS discs was evaluated to assess their suitability for cardiovascular stents and confirm their compatibility with the human body. Tests included cytotoxicity assays, hemolysis ratio measurements, and blood clotting time analysis.

For cytotoxicity tests, mouse fibroblast cells (L929) were used, and the cell culture procedure was performed as described in in previous work.^46^ L929 cells were seeded into well plates at a density of 15 × 10^4^ cells/mL, with 100 μL of cell suspension added to each well, and incubated at 37 °C for 24 h. Simultaneously, extraction liquids were prepared by incubating the test materials (gel samples at 0.2 g/mL), a high-density polyethylene (HDPE) negative control (0.2 g/mL), and a zinc diethyldithiocarbamate (ZDEC) positive control (0.1 g/mL) with medium in the well plate. Following the initial incubation, the medium was aspirated from each well and replaced with 1 mL of the prepared extract, followed by another 24 h incubation at 37 °C. To assess viability, 0.051 mL of XTT reagent was introduced to each well, and the plate was incubated for an additional 3 h at 37 °C. The resulting absorbance of the liquids was measured at 450 nm using a SpectraMax M5 instrument. Cell viability was subsequently determined by calculating the ratio of the change in absorbance for the sample (sample absorbance minus medium absorbance) to the change in absorbance for the blank control (blank absorbance minus medium absorbance).

For the hemolysis test, human whole blood was diluted with PBS to obtain a concentration where a 200 μL aliquot mixed with 3.33 mL of PBS gave an OD of 0.7–0.8 at 542 nm. For the assay, the discs were immersed in 3.33 mL of PBS. DI water (3.33 mL) served as the positive control, while 3.33 mL of PBS was used as the negative control. Next, 200 μL of the diluted blood was added to each tube and incubated at 37 °C for an hour. Following incubation, the solutions were centrifuged at 1200 rpm for 10 min, and 200 μL of the supernatant was collected. Hemoglobin released from lysed red blood cells was quantified by measuring absorbance at 542 nm using a UV–visible spectrophotometer. The hemolysis ratio was calculated according to eq (2).

2 $$Hemolysis\;\left(\%\right)=\frac{{Sample\;OD}_{542}-{PBS\;OD}_{542}}{{DI\;water\;OD}_{542}-{PBS\;OD}_{542}}\times 100\%$$

For blood clotting time analysis, human whole blood was centrifuged at 3000 rpm for 10 min, and the supernatant was collected to obtain platelet-poor plasma (PPP). The SS discs were immersed in 1 mL of PPP and incubated in a water bath at 37 °C for 1 h. After incubation, prothrombin time (PT) and activated partial thromboplastin time (APTT) were measured using a blood clotting time analyzer (Sysmex CA-600 series).

## Results and Discussion

The chemical structures of the zwitterionic monomer and corresponding copolymers are shown in Figure 1. To confirm the successful synthesis of the zwitterionic monomer through the ring-opening addition reaction between 4VP and 1,3-PS, the product was analyzed by ^1^H NMR, as presented in Figure 1a. The spectrum exhibits distinct resonance signals assigned to the vinyl protons (*a*, *b* and *c*) located in the range of 6 – 7.3 ppm,^70^ pyridine ring (*d* and *e*) at 8.2 and 8.9 ppm, and sulfobetaine groups (*f*, *g* and *h*) at 2.6, 3.1, and 4.8 ppm, verifying the successful formation of the zwitterionic monomer.^71^ After polymerization (Figure 1b), the disappearance of the vinyl proton peaks in VPS0 confirmed the successful polymerization of the 4VPPS monomer, while the resonance signals at 2.0 – 2.2 ppm were attributed to the −CH_2_ and −CH (*a’* and *c*) groups in the polymer backbone.^71^ For VPS100 (VPA/4VPPS: 100/0 mol %), the signal (*a*) observed at 1.8–2.2 ppm corresponds to the – CH_2_ groups in the polymer backbone, whereas signal (*b*) is attributed to the methine group of VPA.^72,73^ As for VPS70, signals *a* and *b* were assigned to the VPA segments, while signals *a′* and *c – h* were attributed to the 4VPPS segments, confirming the successful copolymerization through a conventional radical polymerization method.

**Figure 1:**
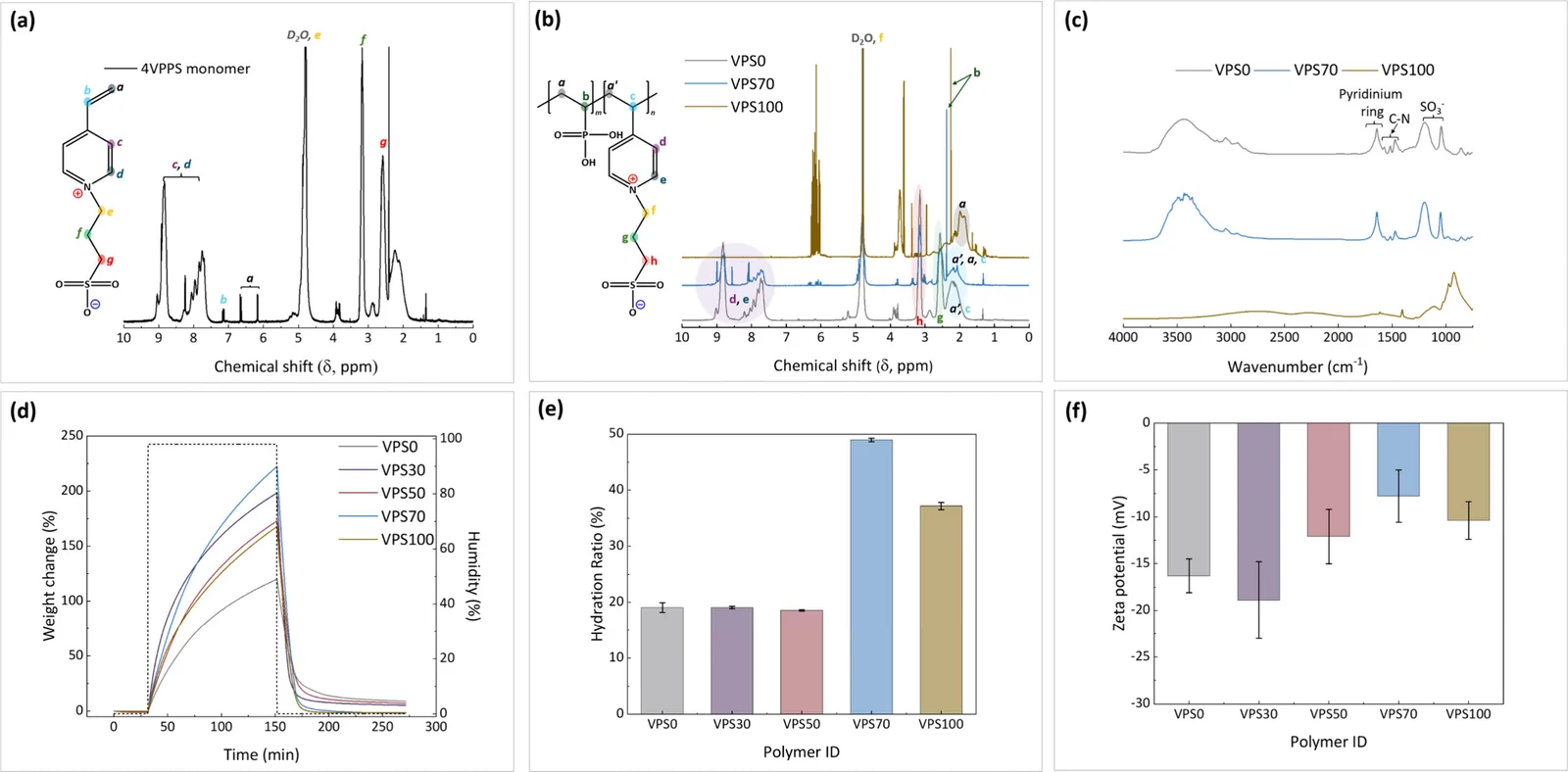
Monomer and copolymer characterization: (a) ^1^H NMR of 4VPPS monomer; (b) ^1^H NMR of P­(VPA-*co*-4VPPS) copolymer; (c) FTIR of P­(VPA-*co*-4VPPS) copolymer; and (d) Dynamic vapor absorption of P­(VPA-*co*-4VPPS) copolymer.

The theoretical and actual molar compositions of the copolymers are summarized in Table 1. Based on the ^1^H NMR analysis, VPS70, VPS50, and VPS30 contained 37.8, 29.6, and 20.1 mol % PVPA, respectively, which are lower than its theoretical values of 70, 50, and 30 mol %. The lower resulting PVPA can be attributed to the inherently lower radical polymerization reactivity of VPA. Its vinyl group is directly connected to a phosphonic acid moiety, which is sterically demanding and highly polar. This bulky pendant group can create steric repulsion near the propagating radical center, thereby reducing the ease of monomer addition during chain growth.^73–75^ In addition, although the phosphonic acid group is electron-withdrawing, it does not provide effective resonance stabilization to the propagating radical because the phosphorus center does not participate in continuous π-conjugation with the vinyl group.^75^ As a result, radical addition to VPA is less favorable compared with more reactive vinyl monomers. Additionally, the strong intermolecular hydrogen bonding generated by its phosphonic acid groups also contributes to its lower reactivity. The P = O and P–OH groups can interact with neighboring VPA molecules, such hydrogen bonding can restrict monomer mobility, increase local viscosity, and reduce availability of properly oriented vinyl groups for radical propagation.^76^ Previous study has also reported that VPA polymerization may involve a higher tendency toward regioirregular addition, such as head-to-head and tail-to-tail sequences. These irregular additions can generate sterically hindered chain ends, further slowing propagation.^75^ Therefore, the lower PVPA content observed in the final copolymers is consistent with the relatively low reactivity of VPA during copolymerization. This resulted in fewer phosphonic acid groups and a higher relative proportion of zwitterion units than expected from the original composition. A lower phosphonic acid content may reduce the number of available anchoring sites on the SS surface. However, this does not necessarily lead to weak attachment, because the remaining phosphonic acid groups can form strong mono-, bi-, or multidentate P–O-M interactions with the surface metal oxides, which may still provide sufficient interfacial stability for durable grafting.

A *M*_w_ of approximately 50 kDa was targeted for all polymer conditions. Previous work on sulfobetaine polymers showed that polymers within this molecular-weight range remained relatively small and well dispersed in aqueous solution, whereas increasing *M*_w_ altered their hydrodynamic size and hemocompatibility.^44^ Although a different sulfobetaine polymer was used in that study, the findings provided a useful basis for selecting a moderate *M*_w_ that could maintain solution mobility while still providing a sufficient number of functional groups along each chain. For the present copolymers, this was expected to facilitate transport toward the SS surface while providing multiple phosphonic acid groups for attachment and zwitterionic units for hydration. The selected value was therefore used as a practical *M*_w_ target rather than an established optimum for P­(VPA-*co*-4VPPS). The experimentally determined *M*_w_ of the polymers and their corresponding PDI are provided in Table 1. The low *M*_w_ of VPS100 could be attributed to its low reactivity. In contrast, *M*_w_ increased progressively as the 4VPPS fraction increased, suggesting that the contribution of VPA-related propagation limitations decreased as the zwitterionic comonomer became more dominant. The broad PDI can be attributed to the limited *M*_w_ control of conventional free-radical copolymerization.^77^ Figure 1c presents the FT-IR spectra of VPS0, VPS70, and VPS100. Strong absorption bands observed at approximately 924 and 972 cm^–1^ are attributed to the stretching vibrations of the P–OH groups, while the broad band centered at 1107 cm^–1^ corresponds to the P = O stretching vibration,^78,79^ all characteristic peaks of phosphonic acid. For VPS0 and VPS70, the peaks centered at 1034 and 1193 cm^–1^ are attributed to the stretching vibrations of S = O and S–O in the SO_3_^–^ groups. The broad band at 1640 cm^–1^ corresponds to the pyridinium ring stretching, while the peak at 1460 cm^–1^ is assigned to the C–N stretching vibration.^70,71^

Figure 1d shows the dynamic vapor sorption profiles of the polymers during a humidity cycle from 0% to 98% RH and back to 0% RH. The humidity was kept at 0% for the first 30 min, followed by an increase to 98% and maintained until 150 min, then returned to 0%. The resulting weight change (%) of each samples indicates the amount of water absorbed and desorbed over time, and their corresponding hydration ratios calculated using eq (2), are provided in Figure 1e. During the high RH stage, all samples showed a significant increase in weight due to moisture absorption. In VPS100, the phosphonic acid groups promoted strong interaction with water under humid conditions. A previous study by Kaltbeitzel et al. showed that PVPA is highly hygroscopic and absorbs increasing amounts of water as the RH rises.^80^ In their work, the water content of PVPA increased progressively with humidity, reaching approximately 0.8 water molecules per phosphonic acid group at 40% RH, while exposure to higher humidity induced substantial swelling and gel formation. A similar humidity-dependent affinity for water may explain the pronounced weight increase observed for VPS100. It exhibited a 167% increase in weight after 150 min, corresponding to a hydration ratio of 37.2%. However, VPS100 rapidly released nearly all of the sorbed water when the RH returned to 0%, indicating that the water uptake was largely reversible.

In contrast, VPS0 exhibited a 120% weight gain at 150 min (hydration ratio of 19.1%), attributed to the formation of a hydration layer through electrostatic interactions between the quaternary ammonium (positive) and sulfonate (negative) groups of the zwitterionic units.^81^ This configuration promotes the formation of a tightly bound and highly ordered hydration shell rather than bulk water clustering. Thus, even after exposure to 0% RH for 200 min, VPS0 retained approximately 13% residual weight, indicating the presence of strongly bound water molecules within its stable hydration layer.

Meanwhile, the copolymers (VPS30, VPS50, and VPS70) exhibited a synergistic hydration behavior resulting from the combined presence of zwitterionic and phosphonic acid moieties. The coexistence of these two functional groups enhanced both the strength and extent of water interaction. Consequently, VPS70, VPS50, and VPS30 showed water uptake values of approximately 222%, 172%, and 198%, corresponding to hydration ratios of 48.9%, 18.6%, and 19.0%, respectively. After 150 min, when the RH was returned to 0%, all samples showed different desorption behaviors. VPS70, despite having the highest water uptake, released almost all of the sorbed water and returned close to its initial weight, which may be related to its lower P4VPPS mol %. In contrast, VPS50 and VPS30, which contained higher P4VPPS fractions, retained higher residual weights after desorption.

Figure 1f displays the zeta potential of the copolymers. VPS100 exhibited a negative zeta potential of −10.4 mV which could be attributed due to the presence of phosphonic acid groups. Although VPS0 consists entirely of zwitterionic P4VPPS, it exhibited a negative zeta potential of −16.3 mV. In the P4VPPS structure, the sulfonate group is positioned at the end of the propyl spacer, whereas the quaternary ammonium group is located closer to the polymer backbone. This spatial separation may make the sulfonate groups more accessible to the surrounding aqueous phase and allow them to contribute more strongly to the electrokinetic potential at the slipping plane. Similar spacer-dependent effects on the zeta potential and hydration of polybetaine materials have been reported.^82^ For the copolymers, VPS50 and VPS30 exhibited zeta potentials of −12.1 and −18.9 mV, respectively, whereas VPS70 showed the least negative value of −7.8 mV.

Developing green and organic-solvent-free surface modification strategies is essential for advancing the fabrication of medical devices. In this work, a water-based grafting method was employed to improve the biocompatibility of SS surfaces while avoiding toxic solvents and harsh reaction conditions. As illustrated in Figure 2a, the copolymer was immobilized onto the SS through a simple dipping process. The phosphonic acid groups formed covalent bonds, likely bi- or multidentate M–O-P linkages,^57,60^ with the surface metal oxides to ensure strong and stable attachment, while the zwitterionic 4VPPS segments extended outward to form a hydrated layer that helps inhibit protein and cell adhesion. This green modification process, performed entirely in aqueous solution under mild conditions, provides a sustainable and cost-effective approach for producing bioinert SS surfaces.

**Figure 2:**
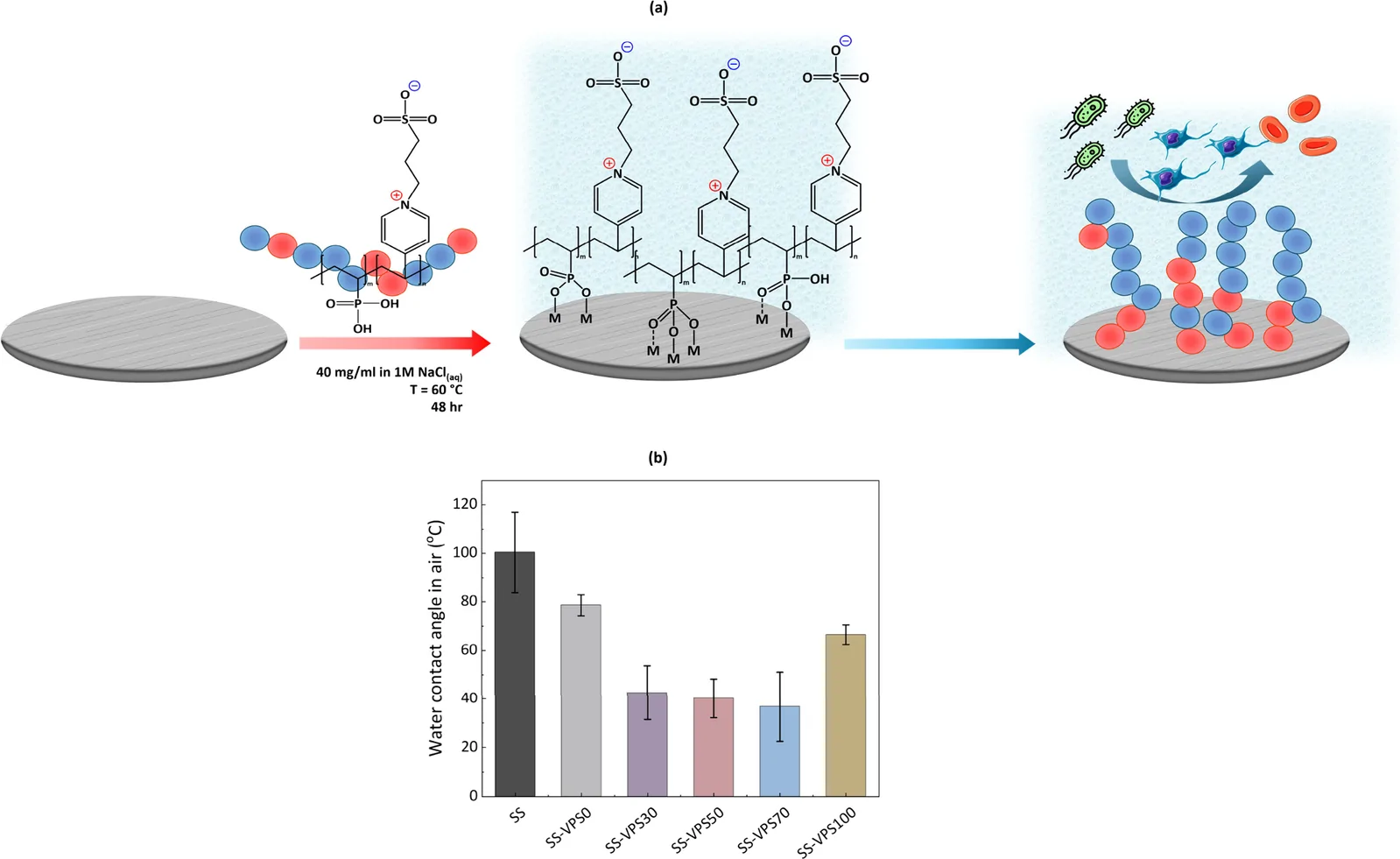
(a) Grafting mechanism of copolymer onto SS surface and (b) WCA evaluation of the SS surface before and after gr.

The surface wettability of the SS samples: SS (virgin) and SS-VPSx (modified) was evaluated by WCA measurements (Figure 2b). The virgin SS exhibited the highest WCA of 100.4°.

When grafted with VPS100, the WCA dropped to 66.6°, indicating enhanced hydrophilicity due to hydrogen bonding between water molecules and the phosphonic acid groups, consistent with the trend observed in Figures 1d-e. The SS-VPS0 surface also showed a reduced WCA of 78.7°, but its higher value compared to VPS100 can be attributed to the absence of phosphate anchoring groups, which likely limited grafting and uniformity. In contrast, the copolymer-modified surfaces (SS-VPS30, SS-VPS50, and SS-VPS70), containing both PVPA anchoring sites and P4VPPS zwitterionic segments, exhibited the lowest contact angles. The phosphate groups facilitated stable chemical bonding with the metal surface, while the outward-oriented zwitterionic segments improved surface hydration, leading to significantly enhanced wettability.

Vascular stents face persistent challenges such as dependence on antithrombotic drugs, in-stent restenosis caused by blood cell and endothelial adhesion, and the risk of infection following implantation. To evaluate the resistance of the grafted surfaces to nonspecific biological attachment, adhesion tests were performed using human fibrosarcoma cells (HT1080), *GFP* *E. coli* as a bacterial model, and undiluted human whole blood. Representative fluorescence images and the corresponding adhesion quantifications are presented in Figure 3.

**Figure 3:**
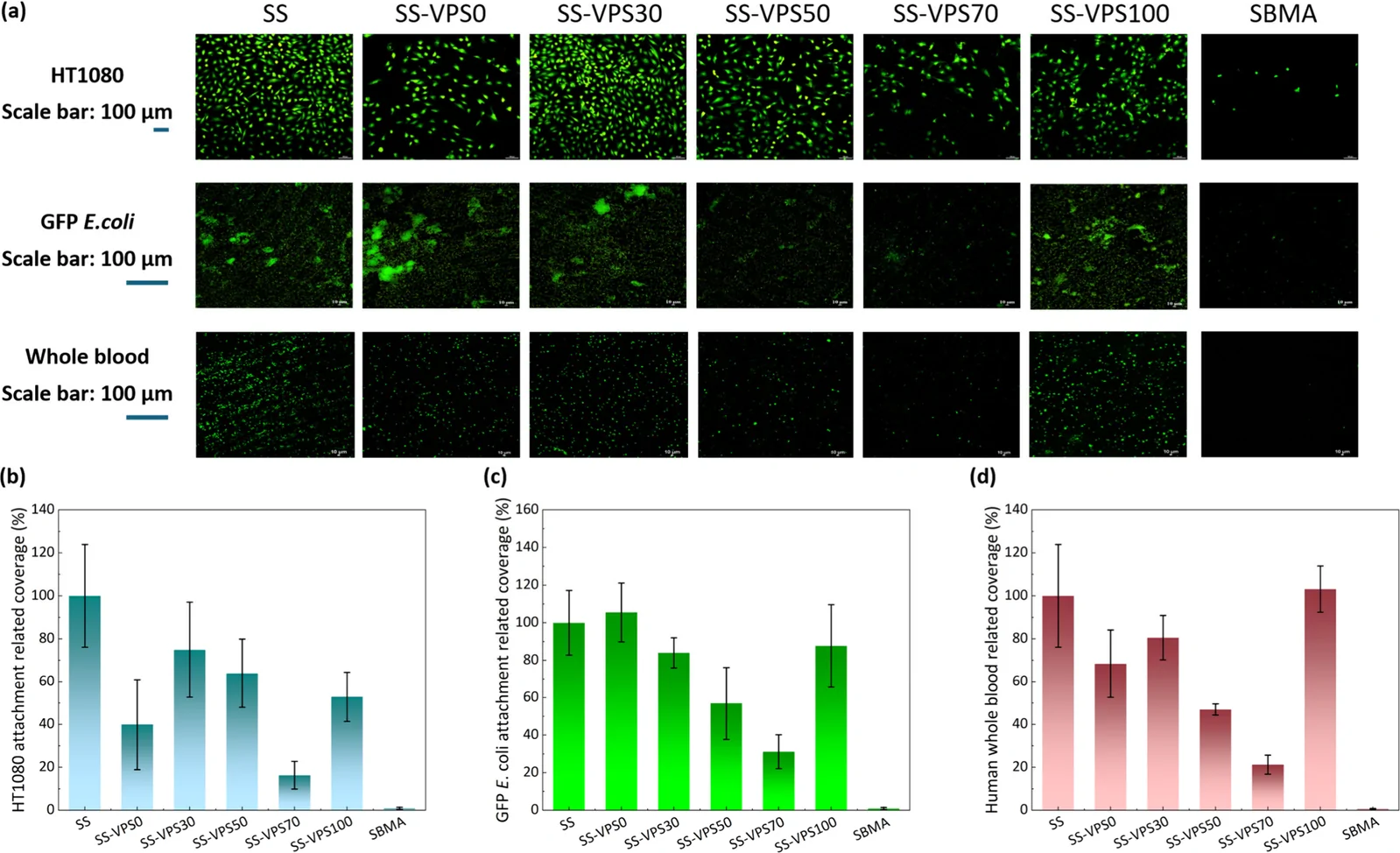
Bioinert evaluation: (a) Fluorescence images of adhesion tests for HT1080, GFP *E. coli*, and human whole blood. (b) Relative coverage area of HT1080 cells. (c) Relative coverage area of GFP *E. coli*. (d) Relative coverage area of human whole blood.

The virgin SS surface exhibited dense coverage of HT1080 fibroblast cells, bacterial aggregates, and blood residues, demonstrating its susceptibility to biological attachment.^83–85^ SS-VPS0 exhibited variable adhesion results, which may be related to less effective or less uniform immobilization because VPS0 lacks phosphonic acid anchoring groups. Although VPS100 showed substantial water uptake and improved wettability, SS-VPS100 provided only moderate resistance to biological adhesion. This may reflect a competition between the water-interacting and surface-anchoring roles of the phosphonic acid groups. Phosphonic acid groups involved in M–O-P linkages may be less available to interact with water at the outer interface, which could limit the formation of an effective hydration layer and contribute to variability in biological adhesion. Similar multifunctional surface-modification strategies commonly separate the anchoring and hydration functions by using phosphonic acid units for attachment to metal oxides and distinct hydrophilic segments to provide resistance to biological adsorption.^86^ Accordingly, the absence of a separate hydration-promoting segment in VPS100 may partly explain why its high bulk water uptake did not translate directly into strong bioinert performance.

A clear improvement was observed across the copolymer series from SS-VPS30 to SS-VPS50 and SS-VPS70. For HT1080 cells, the relative coverage decreased from approximately 75% for SS-VPS30 to 64% for SS-VPS50 and 16% for SS-VPS70. A similar pattern was observed for *GFP* *E. coli*, decreasing from approximately 84% to 57% and 31%, respectively, and for whole blood, decreasing from approximately 80% to 47% and 21%. The similar trend across the 3 biological models suggests that the performance of SS-VPS70 was not specific to a single foulant. Instead, it indicates that VPS70 provided a more generally bioinert surface capable of reducing the attachment of different biological components. This is particularly relevant for blood-contacting devices, where the surface is exposed to multiple foulants simultaneously. SBMA hydrogel was used as the negative control for all of the analyses.

The superior performance of SS-VPS70 can be related to the balance between its phosphonic acid and zwitterionic segments. Although VPS70 was synthesized using a theoretical VPA:4VPPS molar ratio of 70:30, its actual composition was approximately 37.8:62.2, likely because of the lower incorporation of VPA during copolymerization. Nevertheless, the remaining VPA fraction appeared sufficient to support immobilization on the SS surface, as phosphonic acid groups can form multidentate M–O-P interactions with surface metal oxide. At the same time, the higher proportion of 4VPPS provided abundant zwitterionic groups for interfacial hydration. The results therefore suggest that an actual VPA:4VPPS composition close to 40:60 provided a favorable balance between surface attachment and bioinert hydration, which may explain the consistently low adhesion of mammalian cells, bacteria, and whole-blood components on SS-VPS70.

Sterilization is an essential step in medical device preparation to ensure patient safety, with steam sterilization (121 °C, 1 h) being the most commonly used method due to its efficiency and cost-effectiveness.^87^ However, the combined effects of heat and moisture may alter polymer-modified surfaces, particularly materials containing hydrolysis-sensitive ester linkages such as methacrylate-based structures often suffer from hydrolytic degradation and loss of bioinert functionality after exposure to high temperature and moisture.^45,46^

Thermal stability was evaluated by comparing the blood antiadhesion performance of SS-VPS70, and PSBMA hydrogel before and after steam sterilization, as shown in Figure 4. Before sterilization, SS-VPS70 and the PSBMA hydrogel exhibited low relative whole-blood coverages of approximately 21.2% and 0.6%, respectively, compared with the virgin SS reference. Following steam sterilization, the relative whole-blood coverage of SS-VPS70 remained nearly unchanged at approximately 20.7%, and no statistically significant difference was observed between the sterilized and unsterilized surfaces (*p* > 0.05). This preservation of low blood adhesion indicates that the bioinert function of SS-VPS70 was maintained after exposure to 121 °C for 1 h, demonstrating good functional stability under the applied sterilization conditions.

**Figure 4:**
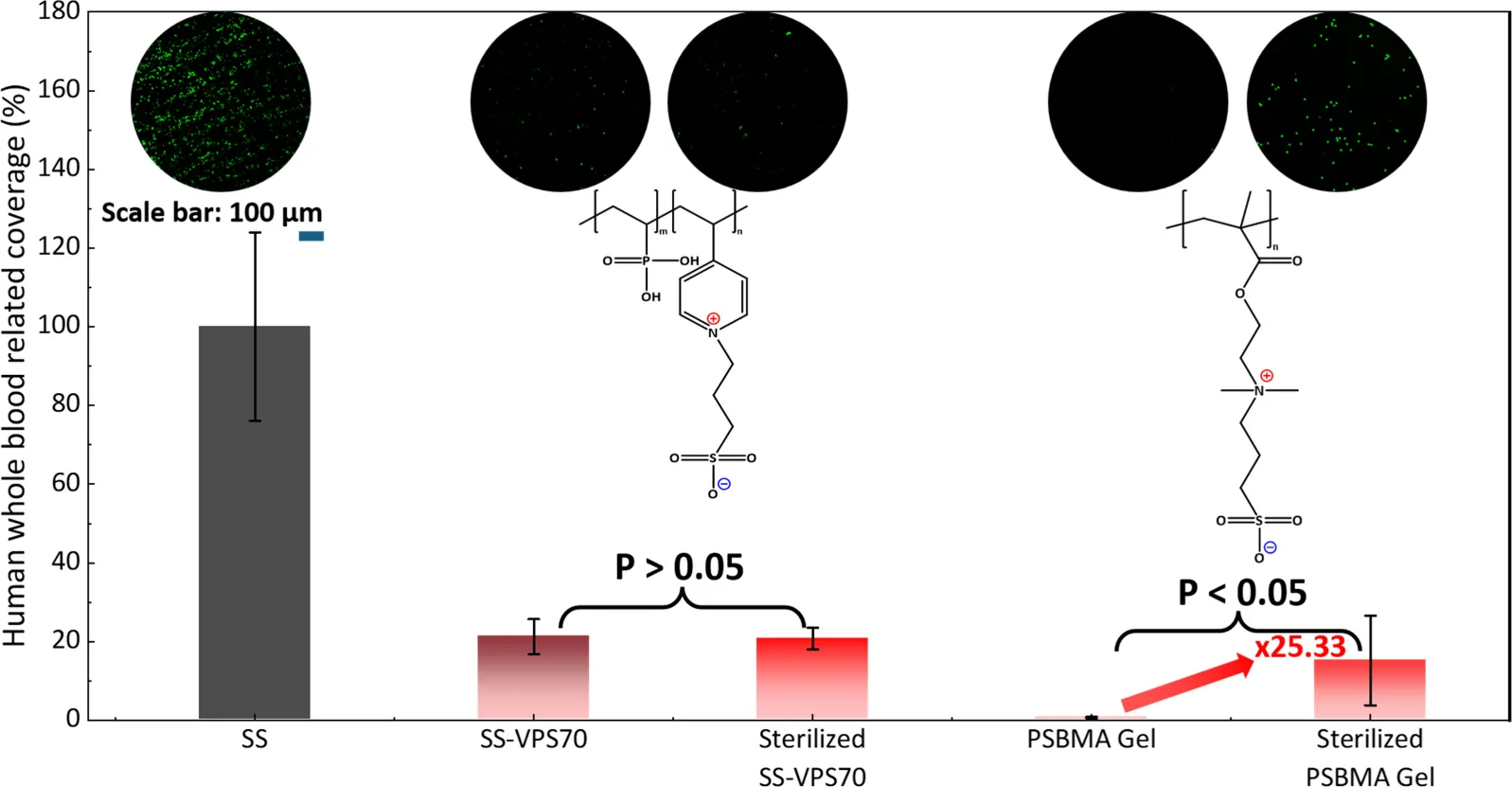
Whole-blood adhesion on unmodified and modified SS surfaces before and after steam sterilization at 121 °C for 1 h. Representative fluorescence images and the corresponding relative surface coverage are shown for each condition.

In contrast, the relative whole-blood coverage of the PSBMA hydrogel increased from approximately 0.6% to 15% after steam sterilization, indicating a significant reduction in its blood-resistant performance. A similar loss of antifouling function after steam sterilization was observed in our previous work on SBMA-containing polymer coatings.^45^ LC-MS analysis revealed cleavage of the ester linkage in SBMA and fragmentation of the polymer after sterilization, which was associated with a marked decline in antifouling performance.

The functional stability of P4VPPS could be attributed to its ester-free structure, in which the sulfobetaine side chain is attached to the pyridinium unit through an N–C bond rather than a hydrolysis sensitive methacrylate ester linkage. Additionally, a previous study^88^ has shown that P4VP and its quaternized derivatives, examined by DSC under inert and oxidative conditions, showed only minor loss around 150 °C, mainly due to the release of adsorbed water. Meanwhile, they denoted that the primary degradation peaks for P4VP and its quaternized forms appeared at approximately 365 and 420 °C, respectively, indicating excellent thermal stability. Similarly, PVPA has been shown to exhibit high thermal resistance, with the first irreversible fragmentation occurring at about 350 °C due to P–C bond cleavage.^80^ Therefore, coupling the thermal stability of these two components enhances the resistance of the grafted copolymer chains to structural degradation under humid heat conditions. The preservation of bioinert performance after steam sterilization addresses a key practical limitation of many zwitterionic surface treatments. This functional durability strengthens the potential of SS-VPS70 as a surface modification for SS biomedical devices that must withstand sterilization before clinical use.

Aside from achieving bioinertness, ensuring biocompatibility is equally critical for cardiovascular stents. A stent must not only resist nonspecific adhesion of cells and proteins but also maintain compatibility with surrounding tissues and blood components to prevent inflammatory or cytotoxic responses. Therefore, the biological safety of the modified surfaces was further assessed through cytotoxicity, hemolysis, and coagulation tests, which provide essential insight into their suitability for long-term use in blood-contacting biomedical devices (Figure 5).

**Figure 5:**
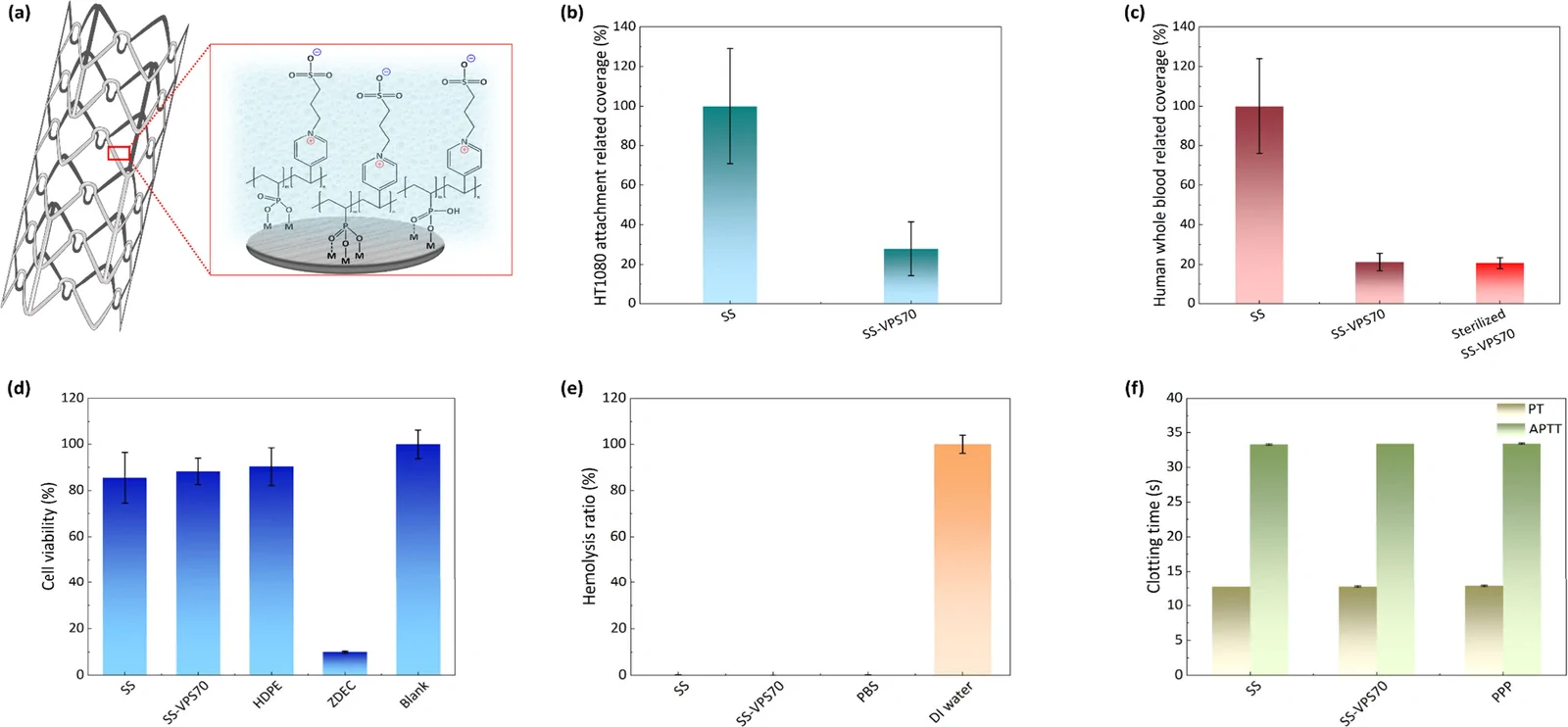
Assessment of the biocompatibility of SS surfaces (virgin and modified). (a) Schematic illustration of VPS70 grafting onto a vascular stent; comparison of bioinert performance between SS and SS-VPS70 in (b) HT1080 cell adhesion and (c) whole blood adhesion tests; (d) cytotoxicity evaluation; (e) hemolysis assay; and (f) blood coagulation analysis.

Figure 5a shows the schematic representation of a VPS70-grafted SS stent, illustrating the potential application of the grafted surface in blood-contacting devices. Figures 5b-c highlight the bionert performance achieved with optimum VPS70 grafting composition (as mentioned previously). The cytotoxicity evaluation (Figure 5d) showed high cell viability for both SS (85.5%) and SS-VPS70 (88.3%), confirming that the grafting did not elicit any cytotoxic response. Figure 5e shows the hemolysis test results, where both SS and SS-VPS70 exhibited 0% hemolysis, consistent with the PBS control and well below the hemolytic threshold, indicating no red blood cell damage and excellent blood compatibility. Coagulation analysis using platelet-poor plasma (Figure 5f) further demonstrated that the prothrombin time (PT) and activated partial thromboplastin time (APTT) values of SS-VPS70 were nearly identical to those of the control group, suggesting that the coating did not interfere with normal clotting processes.

Beyond its biological performance, the VPS70 modification offers a simple and practical route for functionalizing SS surfaces. The copolymer can be applied through a straightforward aqueous process under mild conditions, without organic solvents, complex equipment, or multistep surface treatments. Within the same polymer structure, the phosphonic acid groups provide affinity toward the SS oxide layer, while the zwitterionic segments impart the bioinert and biocompatibility functionalities. This combination of simple processing, water-based chemistry, and dual anchoring-hydration functionality supports the practical adaptation of VPS70 to SS biomedical devices. Building on these findings, future work may evaluate the modified surfaces under dynamic blood-flow conditions and longer exposure periods, while extending the same grafting strategy to other device geometries requiring resistance to biological adhesion and compatibility with steam sterilization.

## Conclusions

In this study, zwitterionic copolymers composed of VPA and 4VPPS were successfully synthesized and grafted onto SS surfaces via a green, water-based approach. Among the copolymers, VPS70 exhibited the most favorable hydration behavior, moderate surface charge, which collectively resulted in superior antifouling and bioinert performance. The VPS70-grafted SS surface effectively suppressed adhesion of fibroblast cells, *E. coli*, and blood components, while maintaining excellent cytocompatibility, nonhemolytic behavior, and normal coagulation behavior. Importantly, its resistance to whole-blood adhesion was preserved after steam sterilization at 121 °C for 1 h, demonstrating functional stability under clinically relevant sterilization conditions. These combined properties, together with the simplicity of the aqueous grafting process, support the potential of VPS70 as a surface modification for cardiovascular stents and other blood-contacting SS devices.
